# Black soldier fly larvae oil (*Hermetia illucens* L.) calcium salt enhances intestinal morphology and barrier function in laying hens

**DOI:** 10.1016/j.psj.2024.103777

**Published:** 2024-04-27

**Authors:** Muhsin Al Anas, Muhammad Anang Aprianto, Henny Akit, Muhlisin, Asih Kurniawati, Chusnul Hanim

**Affiliations:** ⁎ Department of Animal Nutrition and Feed Science, Faculty of Animal Science, Universitas Gadjah Mada, Yogyakarta 55281, Indonesia; † Department of Animal Science, Faculty of Agriculture, Universiti Putra Malaysia, Serdang 43400, Malaysia

**Keywords:** black soldier fly larvae oil, calcium salt, intestinal barrier function, immune response, laying hen

## Abstract

This study aimed to determine the influence of black soldier fly larvae oil calcium salt (**BSFLO-SCa**) supplementation on performance, jejunal histomorphology and gene expression of tight junctions and inflammatory cytokines in laying hens. A total of 60 ISA Brown laying hens (40 wk of age) were divided into 3 treatment groups, including a control group fed a basal diet (**T0**) and basal diets supplemented with 1% (**T1**) and 2% (**T2**) of BSFLO-SCa. Each treatment group consisted of 5 replicates with 4 laying hens each. Results showed that 1% and 2% BSFLO-SCa supplementation significantly reduced (*P* < 0.05) feed conversion ratio (**FCR**), while egg weight (**EW**) increased (*P* < 0.05). The inclusion with 2% increased (*P* < 0.05) both egg production (**HDA**) and mass (**EM**). The addition of 1% and 2% BSFLO-SCa significantly increased (*P* < 0.05) villus height (**VH**) and villus width (**VW**), while crypt depth (**CD**) significantly increased (*P* < 0.05) with 2% BSFLO-SCa. The tight junction and gene expression of claudin-1 (**CLDN-1**), junctional adhesion molecules-2 (**JAM-2**), and occludin (**OCLN**) were significantly upregulated (*P* < 0.05) with 2% BSFLO-SCa. The pro-inflammatory cytokines and gene expression of interleukin-6 (**IL-6**) was significantly downregulated (*P* < 0.05) with the addition of BSFLO-SCa, while gene expression of interleukin-18 (**IL-18**), toll-like receptor 4 (**TLR-4**), and tumor necrosis factor-α (**TNF-α**) were downregulated with 2% BSFLO-SCa. On the other hand, the anti-inflammatory cytokines and gene expression of interleukin-13 (**IL-13**) and interleukin-10 (**IL-10**) were significantly upregulated (*P* < 0.05) at 2% BSFLO-SCa. In conclusion, dietary supplementation with 2% BSFLO-SCa improved productivity, intestinal morphology and integrity by upregulating tight junction-related protein of gene expression of laying hens. In addition, supplementation with BSFLO-SCa enhanced intestinal immune responses by upregulating anti-inflammatory and downregulating pro-inflammatory cytokine gene expression.

## INTRODUCTION

The primary objective of the poultry industry is to guarantee a consistent provision of eggs and meat to meet the needs of consumers. Poultry eggs serve as a crucial and cost-effective source of animal protein (Kumar et al., 2021). The consumption of poultry meat and eggs has doubled in the past decade, driven by rapid growth and fundamental needs. Consequently, the poultry industry has made substantial efforts to address the heightened demand from consumers (Wang et al., 2022). To fulfill the demands, there has been an immense improvement in chicken genetics in the past decade. Laying hen has been selected for higher metabolic rates and egg production (Tallentire et al., 2016), resulting in more generated body heat and susceptible to heat stress. The likelihood of heat stress is heightened with a high stocking density of birds, particularly when combined with elevated ambient temperatures (Goo et al., 2019).

Heat stress represents a significant contributor to overall oxidative stress within the system, as it induces a disruption in the balance between pro-oxidants and antioxidants in the redox system (Mishra and Jha, 2019). Oxidative stress triggers lipid peroxidation, protein nitration, DNA damage, and apoptosis (Estévez, 2015). Moreover, Avian pathogenic *Escherichia coli* (**APEC**) produces the endotoxin lipopolysaccharide (**LPS**), which is pro-inflammatory and induces excessive production of reactive oxygen species (**ROS**), causing oxidative stress (Jiang et al., 2019). Finally, heat stress and LPS induced oxidative stress in the intestine cause damage to intestinal cells, leading to disruption of the integrity and permeability of the intestines. This disturbance affects the digestive process, absorption of feed nutrients, and the overall function of the intestinal barrier (Qiao et al., 2018).

Oxidative stress in the intestine is indicated by a production response of reactive species, such as nitric oxide (**NO**), which exhibits antimicrobial activity (Baümler and Sperandio, 2016). The reactive species of NO is secreted into the intestinal lumen, and quickly transforms into nitrate. If left uncontrolled, the population of *Escherichia coli (E. coli*) is expected to increase due to its ability to produce nitrate reductase (Gresse et al., 2017). Consequently, this may result in an increase in the number of pathogenic bacteria and reduction in the number of good bacteria in the digestive tract (Daneshmand et al., 2019). Pathogenic bacterial infections contribute to the activation of immune system cells, induce inflammation, and trigger the excess production of free radicals (Abbas et al., 2020).

The internal mechanisms of the body's immune system in fighting pathogenic infections involve the secretion of pro-inflammatory cytokines, namely interferon gamma (**IFN-γ**), interleukin-2 (**IL-2**), tumor necrosis factor α (**TNF-α**), lymphotoxin, and granulocyte-macrophage colony-stimulating factor, which are mediated by T helper cells (**Th1**) (Raphael et al., 2015). The secretion of pro-inflammation cytokines, and the excessive formation of free radicals due to infection indicate that pathogens have the potential to cause tissue damage (Abbas et al., 2020). The same mechanism also occurs in oxidative stress due to heat stress. Heat stress will create pro-inflammatory environment in the body resulting in loss of intestinal integrity. Subsequently, toxins and pathogenic bacteria will enter the lumen of intestines and induce the pro-inflammatory cytokines (Zmrhal et al., 2023). Moreover, villous growth will be disrupted due to heat stress and pathogenic infection which are characterized by impairment of intestinal epithelium integrity, decreasing villus height (**VH**) and VH-to-crypt depth (**CD**) ratio (**VH:CD**) (Li et al., 2018; Mazzoni et al., 2022). A potential approach to reduce the adverse impacts of infections caused by pathogens and heat stress induced oxidative stress involves the use natural of feed additives with antibacterial activity (Oni et al., 2024).

Black soldier fly (**BSF**) larvae, *Hermetia illucens*, are popular in poultry feed due to their high crude protein content (approximately 40.8 ± 3.8%). Moreover, BSF larvae can efficiently process organic waste, making them increasingly popular for development. On the other hand, to produce meal for poultry diet, BSF larvae are extracted and result in black soldier fly larvae oil (**BSFLO**) (Wang and Shelomi, 2017). Recently, many researchers are interested in the fatty acid content of BSLO due to its high concentration of medium-chain fatty acid (**MCFA**), mainly lauric acid (C12:0), as high as 52% of the total fatty acid content (Ewald et al., 2020). Lauric acid has a broad spectrum anti-microbiological activity against Gram-positive bacteria by disrupting the bacterial cell wall or membrane (Fischer et al., 2012). Lauric acid is known to have high antimicrobial activity against pathogenic Bacteroidetes and Clostridium, but a low antimicrobial activity against lactic acid bacteria. As a result, lauric acid can modulate intestinal health (Matsue et al., 2019). Gut health is characterized with good intestinal morphology and tight junction integrity with improved nutrient absorption capacity of the intestine (Wickramasuriya et al., 2022). Moreover, the disruption of the tight junction barrier's structure and integrity can induce inflammation (Lechuga et al., 2023). Many studies reported that supplementation of BSFLO had no impact on production performance of laying hens (Patterson et al., 2021; Kim et al., 2022). Interestingly, BSFLO improved the villus height and gene expression of tight junction of broiler chickens (Chen et al., 2022). To the best our knowledge, there are no studies related to mRNA expression of tight junction and inflammatory factors in laying hens supplemented with BSFLO. Therefore, in this study, the effect of Black Soldier Fly Larvae Oil Calcium Salt (**BSFLO-SCa**) supplementation on intestinal histomorphology, intestinal barrier function, and inflammatory response in laying hens were investigated.

## MATERIALS AND METHODS

All animal procedures of this experiment were approved by the Research Ethics Committee at the Faculty of Veterinary Medicine, Universitas Gadjah Mada No. 00149T/EC-FKH/Ex./2021.

### Preparation of Black Soldier Fly Larvae Oil Calcium Salt and Crude Palm Oil Calcium Salt

Black soldier fly larvae oil in this study was obtained from the PT. Magalarva Sayana Indonesia (Banten, Indonesia), while crude palm oil (**CPO**) was obtained from PT. Sari Rosa Asih (Yogyakarta, Indonesia). The BSFLO-SCa and Crude Palm Oil Calcium Salt (**CPO-SCa**) were prepared by mixing the oil with 10.4% NaOH solution and vigorously stirred until solid. Then, 10.4% CaCl_2_ solution was added and vigorously stirred until yellowish solid particles were obtained according to the method described by (Aprianto et al., 2023). The fatty acid compositions of BSFLO-SCa and CPO-SCa were analyzed by gas chromatography (GC-Agilent Technologies 7980B, California), according to the method presented by Mjøs (2003) with modification. The chromatogram peaks can be identified based on the retention time and compared with commercial standards. Gross energy (**GE**) value both of supplements were analyzed using CAL3K-S oxygen bomb calorimeter system (Digital data system, Randburg, South Africa) following the the method presented by Hopper et al. (2024). The fatty acid compositions and GE value of BSFLO-SCa and CPO-SCa are outlined in Table 1.

**Table 1:** Fatty acid profiles and gross energy value of black soldier fly oil calcium salt (**BSFLO-SCa**) and crude palm oil calcium salt (**CPO-SCa**).

Items	BSFLO-SCa	CPO-SCa
Fatty acid profiles (%)		
Decanoic (C10:0)	1.34	0.79
Lauric (C12:0)	36.96	0.30
Myristic (C14:0)	7.90	1.20
Pentadecanoic (C15:0)	0.20	0.19
Palmitic (C16:0)	15.46	55.20
Heptadecanoid (C17:0)	0.34	0.17
Stearic (C18:0)	nd.	4.50
Heneicosanoic (C21:0)	0.35	nd.
Lignoceric (C24:0)	0.43	0.16
Palmitoleic (C16:1)	2.27	3.35
Oleic (C18:1)	0.44	15.20
Linoleic (C18:2)	34.36	19.82
Gamma-lonoleic (C18:3)	nd.	0.45
Eicosanoic (C20:1)	1.04	nd.
Linolenic (C18:3)	0.23	0.91
Eicosatrienoic (C20:3)	0.18	0.20
Eicosatetranoic (C20:1)	nd.	nd.
Nervoic (C24:1)	nd.	0.15
SFA	62,98	62.51
MUFA	3.71	18.70
PUFA	34.77	21.38
Gross energy (Cal/g)	6,942.69	7,264.40

Abbreviations: BSFLO-SCa, black soldier fly larvae oil calcium salt; CPO-SCa, crude palm oil calcium salt; SFA, Saturated fatty acids; MUFA, Monounsaturated fatty acids; PUFA, Polyunsaturated fatty acids.

### Birds and Experimental Design

A total of 60 Isa Brown (40-wk-old) laying hens with similar body weight (1837.50 ± 21.79) were randomly assigned to 3 treatment groups with 5 replicates in each group and 4 laying hens in each replicate. There was no significant difference on hen-day egg production rate among these groups (86.78 ± 2.68%). The birds were raised in cages with 52 × 35 × 40 cm dimensions with the stocking density of 455 cm^2^ of cage area per bird. The laying hen management followed the recommendations of the Isa Brown Management Handbook (ISA, 2023). The temperature and relative humidity of the poultry house were maintained at 20 ± 2°C and 60 to 65%, respectively. All birds were subjected to a light/dark cycle (16 h light: 8 h dark). The lighting density was set at 15 lux. The experimental diets and drinking water were supplied *ad libitum*. Feed was supplied in troughs placed in front of each cage twice a day, and water was provided from nipple drinkers continuously. The feeding experiment lasted for 8 wk.

### Experimental Design and Diet

This study used a 1-way pattern design (completely randomized design). The birds in each group were fed a basal diet based on CPO-SCa as a control (**T0**) and substituted with 1% (**T1**) and 2% (**T2**) of BSFLO-SCa. The diets were formulated to be isocaloric and isonitrogenous across treatments to meet or exceed the nutrient requirements of laying hens according to ISA (2023) recommendations, as is shown in Table 2. The fatty acid profiles of experimental diets were assessed using gas chromatography (GC-Agilent Technologies 7980B, CA), following the procedure outlined by Mjøs (2003) with adjustments. Identification of chromatogram peaks was done based on retention time and compared against commercial standards. The fatty acid compositions of experimental diets are outlined in Table 3.

**Table 2:** Compositions and nutrient content of experimental diets.

Items	Percentage (%)		
	T0	T1	T2
Feed ingredients			
Corn	48.25	48.55	48.75
Rice bran	5.00	4.70	4.50
Soybean meal	31.99	31.99	31.99
CPO-SCa	2.00	1.00	0.00
BSFLO-SCa	0.00	1.00	2.00
Limestone	10.00	10.00	10.00
Dicalcium phosphate	1.50	1.50	1.50
Sodium chloride	0.36	0.36	0.36
Vitamin Mix^1^	0.06	0.06	0.06
Mineral Mix^2^	0.19	0.19	0.19
DL-Methionine	0.25	0.25	0.25
Choline chloride	0.11	0.11	0.11
Toxin binder	0.19	0.19	0.19
Analyzed nutrients			
Dry matter (%)	89.68	88.73	88.87
Crude protein (%)	19.65	19.35	19.20
Ether extract (%)	3.50	3.70	3.80
Crude fiber (%)	3.85	2.85	3.00
Ash (%)	13.95	14.88	14.45
Calculated nutrients			
Metabolizable energy (Kcal/kg)	3064.19	3064.19	3064.19
Calcium (%)	4.91	4.91	4.91
Available Phosphorus (%)	0.40	0.40	0.40
Methionine (%)	0.61	0.61	0.61
Lysine (%)	1.46	1.46	1.46
Threonine (%)	0.89	0.89	0.88

Abbreviations: CPO-SCa, crude palm oil calcium salt; BSFLO-SCa, black soldier fly larvae oil calcium salt.

1 Supplied per kg of diet: Vitamin A, 50,000,000 IU, Vitamin D3, 10,000,0000 IU; Vitamin E, 80,000 mg; Vitamin K3, 10,000 mg; Vitamin B1, 10,000 mg; Vitamin B2, 30,000 mg, Vitamin B3, 225,000 mg; Vitamin B5, 62,000 mg, Vitamin B6, 10,000 mg; Vitamin B9, 5,000 mg; Vitamin B12, 100 mg; Vitamin H, 100 mg; Vitamin C, 20,000 mg.

2 Supplied per kg of diet: Mn, 50,000 mg; Fe, 30,000 mg; Cu, 7,500 mg; Zn, 40,000 mg; I, 755 mg; Se, 150 mg. T0: basal diet; T1: basal diet + 1% BSFLO-SCa; T2: basal diet + 2% BSFLO-SCa.

**Table 3:** Fatty acid compositions of experimental diets.

Fatty acids	Treatments		
	T0	T1	T2
Lauric (C12:0)	0.50	11.92	13.93
Myristic (C14:0)	0.97	3.29	3.48
Palmitic (C16:0)	39.61	23.03	22.93
Heptadecanoid (C17:0)	0.11	0.19	0.15
Stearic (C18:0)	4.47	5.27	3.83
Docosanoic (C22:0)	0.18	1.38	1.36
Lignoceric (C:24)	0.37	0.96	0.10
Palmitoleic (C16:1)	0.15	0.52	0.83
Oleic (C18:1)	35.80	28.81	27.85
Linoleic (C18:2)	15.94	20.35	22.33
Gamma-linolenic (C18:3)	0.47	0.70	0.52
Eicosanoic (C20:1)	0.73	0.83	0.98
Linolenic (C18:3)	0.20	0.27	0.24
Eicosadienoic (C20:2)	0.10	0.91	0.14
Eicosapentaenoate (C20:5)	0.17	0.37	0.29
Nervoic (C24:1)	0.23	0.52	0.49
SFA	46.21	46.04	45.88
MUFA	38.91	30.68	30.15
PUFA	16.88	22.60	23.52

Abbreviations: BSFLO-SCa, black soldier fly larvae oil calcium salt; SFA, saturated fatty acids; MUFA, monounsaturated fatty acids; PUFA, polyunsaturated fatty acids.

T0: basal diet; T1: basal diet + 1% BSFLO-SCa; T2: basal diet + 2% BSFLO-SCa.

### Laying Performance

Eggs were collected on a daily basis to determine egg weight (**EW**), and egg mass (**EM**)**,** and Hen-day egg production (**HDA**). Egg mass was determined by multiplying of EW by HDA, while HDA was calculated by dividing the total egg production by the total number of hens and then multiplied by 100. Egg production, egg weight, and feed consumption recorded to calculate the avarege daily feed intake (**ADFI**) and feed conversion ratio (**FCR**) of laying hens on a weekly basis. Productive performance data were complied during an 8-week feeding study.

### Jejunal Histomorphology

At the end of the experiment, one bird in each group with a body weight close to the median for each group was selected, resulting in a total of 15 birds, with 5 birds per group. Birds were exsanguinated by cutting the jugular veins and the digestive tracts were removed from the carcass. The middle part of jejunum samples was cut about 2 cm and processed by following the previously published protocol (Li et al., 2022). Briefly, the tissue samples of jejunum were fixed in 10% paraformaldehyde, decalcified in solution of 10% ethylenediaminetetraacetic acid plus 1% sodium hydroxide solution, dehydrated with ethanol, and embedded in paraffin. Each tissue was cut into 3 sections with well-oriented parts using the microtome Leica RM 2235 (Leica Microsystems Ltd., Deer Park, IL), then dewaxed with xylene and stained with hematoxylin and eosin stains (**H&E**). The histomorphology images were taken at 40Χ using microscope (Nikon, Tokyo, Japan). The villus height (**VH**) and crypt depth (**CD**) were measured and recorded by Image-Pro Plus 6.0 software, and the ratio of villus width to crypt depth (**VH/CD**) was calculated.

### Intestinal Tight Junction and Inflammatory Factors Genes Expression in Quantitative Real Time-PCR

Jejunal samples were collected in a microtube from 5 birds in each treatment. Microtubes were instantly frozen in liquid nitrogen and kept at -80°C until analyzed. The gene expression analysis begins with RNA extraction from a 20 mg jejunum sample using a Quick-RNA minirep kit (Zymo Research Corp., Irvine, California) according to instructions by manufacturer. The Nanodrop Spectrophotometer (Maestrogen Inc, Hsinchu City, 30091, Taiwan) was used to determine the purity and amount of RNA. The total RNA was used as a template for cDNA synthesis with ReverTrace qPCR RT Master Mix (Toyobo Co., Ltd., Osaka, Japan). According to the protocol, relative gene expressions were measured using a QuantStudio 3 Real-Time PCR machine (Thermo Fisher Scientific, Waltham, MA) and Thunderbird SYBR qPCR Mix (Toyobo Co., Ltd., Osaka, Japan, Cat No. QPX-201). In a 20 μL reaction volume containing nuclease-free water, 2 μL diluted cDNA, 6 pmol forward primer, 6 pmol reverse primer, 0,04 μL ROX reference dye, and 10 μL qPCR Mix were poured into the qPCR tube (Thermo Fisher Scientific, Waltham, MA).

Table 4. shows the gene-specific primers used for analysis of mRNA levels in this study. The following amplification schedule was used: a hold stage at 95°C for 2 min, followed by a PCR stage at 95°C for 1 s and 60°C for 30 s. The melt curve was examined to identify product amplification at the end of the run. Each group received eight samples, with each sample being conducted in duplicate. The 2^−ΔΔCT^ technique was used to normalize the mRNA levels as a ratio to ß-actin in arbitrary units and the data were reported as relative values to the control group (Livak and Schmittgen, 2001).

**Table 4:** Gene-specific primers used for analysis of mRNA levels of tight junction and inflammatory using quantitative real-time RT-PCR

Gene	Primer sequence (5′->3′)	Orientation	Base Pairs	Reference
β-actin	GTGTGATGGTTGGTATGGGC	Forward	225	Xie et al. (2019)
	CTCTGTTGGCTTTGGGGTTC	Reverse		
CLDN-1	GGTGAAGAAGATGCGGATGG	Forward	137	Proszkowiec-Weglarz et al. (2020)
	ATCGCCCTGTCCGTCATC	Reverse		
OCLN	GATGGACAGCATCAACGACC	Forward	142	Proszkowiec-Weglarz et al. (2020)
	CTTGCTTTGGTAGTCTGGGC	Reverse		
ZO-1	GCCAACTGATGCTGAACCAA	Forward	141	Proszkowiec-Weglarz et al. (2020)
	GGGAGAGACAGGACAGGACT	Reverse		
JAM-2	CTGCTCCTCGGGTACTTGG	Forward	135	Proszkowiec-Weglarz et al. (2020)
	CCCTTTTGAAAATTTGTGCTTGC	Reverse		
TLR-3	GATTGCACCTGTGAAAGCATTG	Forward	67	Xie et al. (2019)
	CGGGTATATATGCTTGAGTGTCGTT	Reverse		
IL-18	TGCAGCTCCAAGGCTTTTAAG	Forward	63	Mullenix et al. (2022)
	CTCAAAGGCCAAGAACATTCCT	Reverse		
IL-6	GCTTCGACGAGGAGAAATGC	Forward	63	Mullenix et al. (2022)
	GGTAGGTCTGAAAGGCGAACAG	Reverse		
TLR-4	TCCTCCAGGCAGCTATCAAGAT	Forward	74	Xie et al. (2019)
	GACAACCACAGAGCTCATGCA	Reverse		
TNF-α	CGTTTGGGAGTGGGCTTTAA	Forward	61	Mullenix et al. (2022)
	GCTGATGGCAGAGGCAGAA	Reverse		
IL-13	CCAGGGCATCCAGAAGC	Forward	256	Fasina and Lillehoj (2019)
	CAGTGCCGGCAAGAAGTT	Reverse		
IL-10	CATGCTGCTGGGCCTGAA	Forward	63	Xie et al. (2019)
		Reverse		

Abbreviations: CLDN-1, Claudin-1; OCLN, Occludin; ZO-1, Zonula occludens-1; JAM-2, Junctional adhesion molecule-2; TLR-3, Toll-like receptor-3; IL-18, Interleukin-18, IL-6, Interleukin-6, TLR-4,Toll-like receptor-4, TNF-α, Tumor necrosis factor-α; IL-13, Interleukin-13; IL-10, Interleukin-10.

### Statistical Analyses

All experimental data were analyzed statistically using IBM SPSS statistic version 26.0. The data subjected to one-way ANOVA. Duncan test was used to determine significant differences among the 3 treatments. The results were considered statistically significant when *P* < 0.05.

## RESULTS

### Laying Hens Performance

The productivity of laying hens with the inclusion of BSFLO-SCa in diet is presented in Table 5. On wk 41 to 44, HDA increased (*P* < 0.05), while feed intake, egg weight, and egg mass showed no differences (*P* > 0.05) with the addition of 2% BSFLO-SCa. Feed utilization efficiency enhanced with the addition of 1% and 2% BSFLO-SCa in diet, resulting better FCR value (*P* < 0.05). On wk 45 to 48, the inclusion with 2% BSFLO-SCa led to significant increase in HDA and egg mass (*P* < 0.05). However, there was no notable variance detected in egg weight and feed intake (*P* < 0.05). The FCR was significantly lower when supplemented with 2% BSFLO-SCa in comparison to both the control group and 1% BSFLO-SCa (*P* < 0.05). Overall (1–8 wk), supplementation of 2% BSFLO-SCa to the diet enhanced productivity (HDA, EM), while FCR decreased with 1% and 2% BSFLO-SCa (*P* < 0.05). The inclusion 1% BSFLO-SCa increased EW in laying hens (*P* < 0.05).

**Table 5:** Effect of supplementation with BSFLO-SCa on performance in laying hens (n = 5 per treatment).

Parameters	BSFLO-SCa			SEM	*P*-value
	T0	T1	T2		
41–44 wk					
HDA (%)	84.30^b^	91.07^ab^	91.51^a^	1.471	0.049
EM (g)	54.85	59.01	58.60	0.946	0.140
EW (g)	62.57^b^	65.21^a^	64.27^ab^	0.463	0.049
ADFI (g)	113.39	108.41	115.86	1.540	0.130
FCR	2.07^a^	1.84^b^	1.86^b^	0.044	0.046
45–48 wk					
HDA (%)	82.54^b^	85.71^ab^	90.41^a^	1.301	0.031
EM (g)	48.37^b^	54.21^ab^	57.99^a^	1.586	0.073
EW (g)	61.95^b^	65.83^a^	64.80^ab^	0.691	0.046
ADFI (g)	110.02	109.89	105.68	1.605	0.489
FCR	2.16^a^	2.15^a^	1.87^b^	0.038	<0.001
41–48 wk					
HDA (%)	83.42^b^	88.39^ab^	90.96^a^	1.198	0.018
EM (g)	52.11^b^	56.61^ab^	58.30^a^	1.091	0.043
EW (g)	62.27^a^	65.52^b^	64.53^ab^	0.546	0.030
ADFI (g)	111.70	109.15	110.77	1.004	0.611
FCR	2.11^a^	2.00^b^	1.87^c^	0.033	0.002

Abbreviations: SEM, standard error of the mean; HDA, hen day average; EM, egg mass; EW, egg weight; ADFI, average daily feed intake; FCR, feed conversion ratio.

T0: basal diet; T1: basal diet + 1% BSFLO-SCa; T2: basal diet + 2% BSFLO-SCa.

a-c Means within a column with different superscripts are different (*P* < 0.05).

### Jejunal Histomorphology

The effect of dietary supplementation of BSFLO-SCa on jejunal histomorphology is presented in Table 6. Dietary supplementation of 1% and 2% BSFLO-SCa increased (*P* < 0.05) jejunal villus height and width, while 2% BSFLO-SCa significantly increased (*P* < 0.05) jejunal crypt depth. There was no significant difference (*P* > 0.05) on the ratio of villus height and crypt depth.

**Table 6:** Effect supplementation of black soldier fly larvae oil calcium salt (**BSFLO-SCa**) on jejunal histomorphology of laying hens (n=5 per treatment).

Parameters	Treatments			SEM	*P*-value
	T0	T1	T2		
Villus height (μm)	1061.85^b^	1475.12^a^	1476.30^a^	61.122	<0.001
Villus width (μm)	209.58^b^	283.09^a^	278.88^a^	14.636	0.047
Crypt depth (μm)	179.87^b^	229.74^ab^	269.18^a^	13.528	0.012
VH:CD	5.10	5.51	5.36	0.274	0.849

Abbreviation: SEM, Standard error of the mean.

T0: basal diet; T1: basal diet + 1% BSFLO-SCa; T2: basal diet + 2% BSFLO-SCa.

a,b,c Means within a column with different superscripts are different (*P* < 0.05).

### Tight Junction Gene Expression

**Figure 1:**
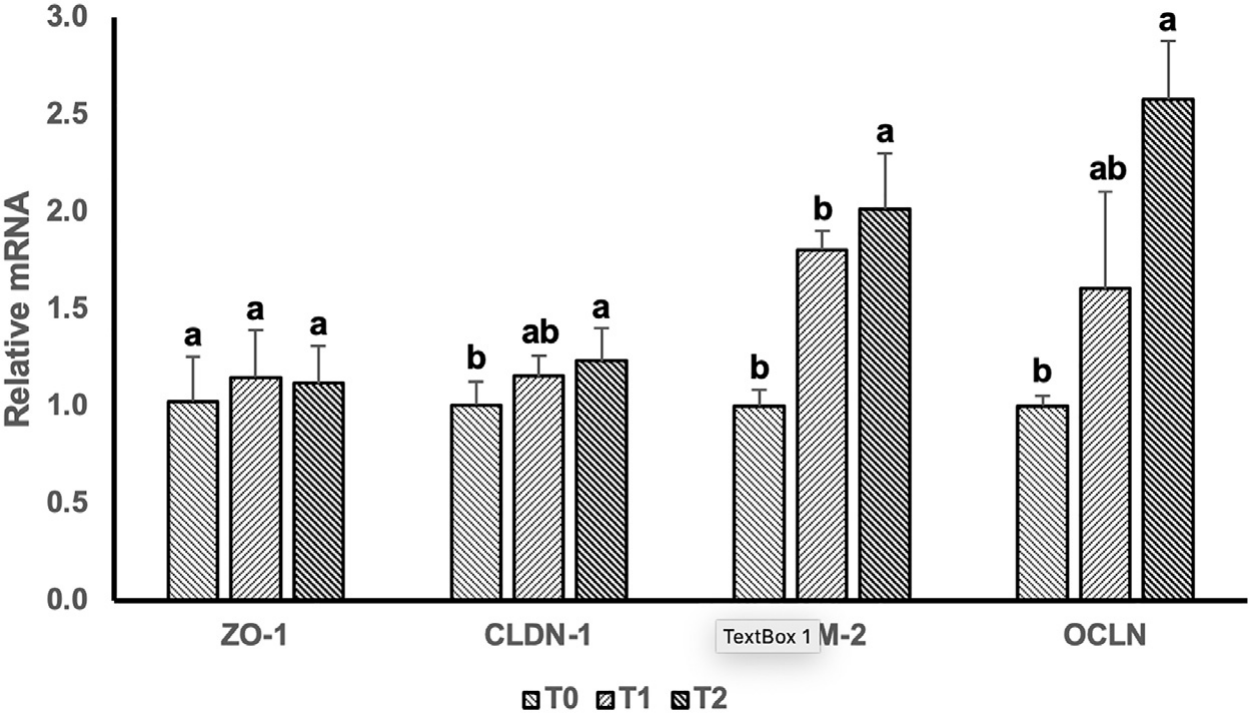
Comparisons between jejunal ZO-1, CLDN-1, JAM-2 and OCLN mRNA expression in laying hens supplemented with black soldier fly larvae oil calcium salt (BSFLO-SCa) at 0% (T0), 1% (T1), and 2% (T3). Data represents the mean value of 5 replicates with 1 bird per replicate. ^a,b^different superscripts on the same target gene showed significant differences (*P* < 0.05). Abbreviations: ZO-1, Zonula occludens-1; CLDN-1, Claudin-1; JAM-2, Junctional adhesion molecule-2; OCLN, Occludin.

The effect of dietary supplementation of BSFLO-SCa on expression of tight junction genes is presented in Figure 1. Birds supplemented with 2% BSFLO-SCa showed increased gene expression of CLDN-1, JAM-2, and OCLN genes compared to the control group (*P* < 0.05). However, the inclusion with 1% BSFLO-SCa no difference in the expression of the CLDN-1, JAM-2, and OCLN compare with control treatments (*P* > 0.05). Furthermore, there was no difference in the expression of the ZO-1 gene between treatments (*P* > 0.05).

### Inflammation Gene Expression

**Figure 2:**
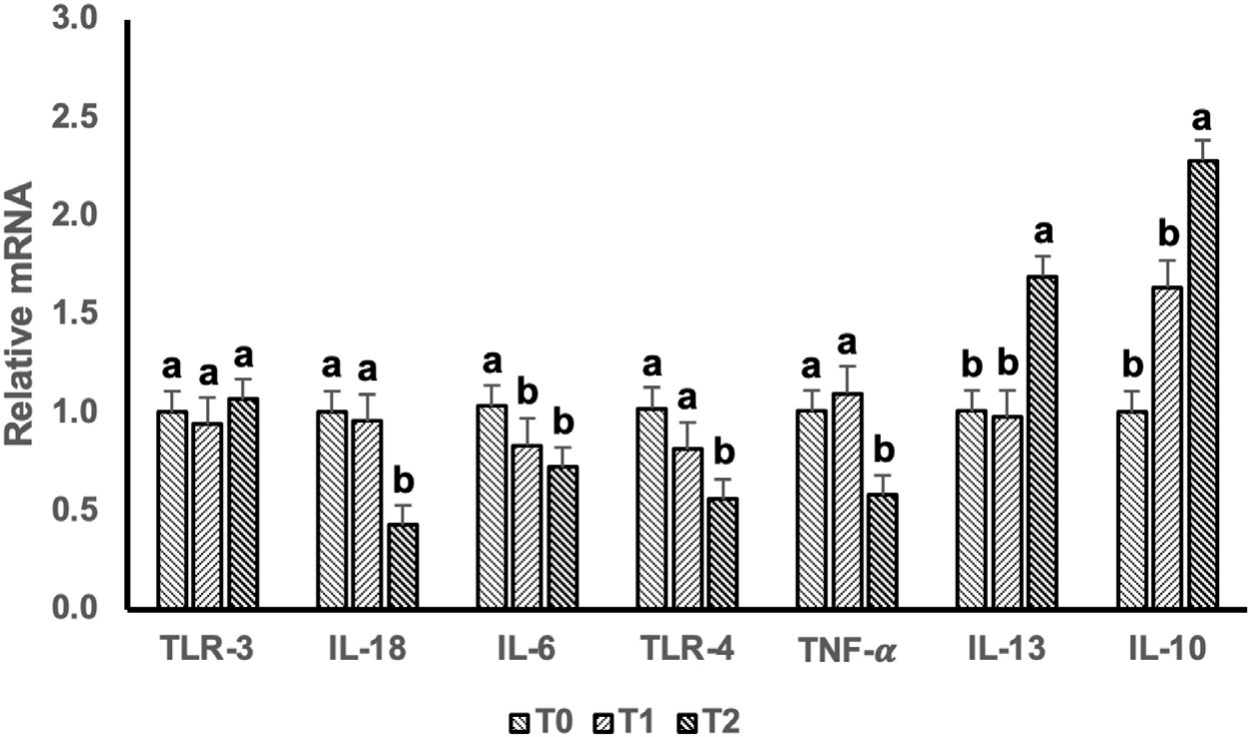
Comparisons between jejunal TLR-3, IL-18, IL-6, TLR-4, TNF-α, IL-13, and IL-10 mRNA expression of laying hens supplemented with black soldier fly larvae oil calcium salt (BSFLO-SCa) at 0% (T0), 1% (T1), and 2% (T3). Data represents the mean value of 5 replicates with 1 bird per replicate. ^a,b^ different superscripts on the same target gene showed significant differences (*P* < 0.05). Abbreviations: TLR-3, Toll-like receptor-3; IL-18, Interleukin-18, IL-6, Interleukin-6, TLR-4, Toll-like receptor-4, TNF-α, Tumor necrosis factor-α; IL-13, Interleukin-13; IL-10, Interleukin-10.

Gene expression related to inflammatory factors in dietary supplementation of BSFLO-SCa is presented in Figure 2. Dietary supplementation with 1% and 2% BSFLO-SCa significantly downregulated (*P* < 0.05) the expression of pro-inflammatory factors; IL-6, while IL-18, TNF-α, and TLR-4 significantly downregulated (*P* < 0.05) with 2% of BSFLO-SCa. Dietary supplementation with 2% BSFLO-SCa upregulated the gene expression of anti-inflammatory factors; IL-13 and IL-10 (*P* < 0.05).

## DISCUSSION

### Laying Hens Performance

In the current study, the inclusion of BSFLO-SCa at 2% in the diet enhanced productivity (HDA, EM), while feed conversion ratio decreased (from 1 to 8 weeks). Research on the utilization of BSFLO in the diet of laying hens is limited. Previous research has examined the utilization of BSFL meal in laying hens (Heuel et al., 2021). Our findings differ with Patterson et al. (2021), who demonstrated that including BSFLO into feed did not impact the performance of laying hens. The difference in findings may be ascribed to the distinct fatty acid composition of BSFLO. The composition of fatty acids can be influenced by the growing media of larvae (Ewald et al., 2020). Differences in fatty acid composition in diet can impact productivity in laying hens (Gao et al., 2021).

Our finding revealed villi and tight junctions of laying hens enhanced with the inclusion of BSFLO-SCa in diet (Table 6 and Figure 1). Furthermore, treatment with BSFLO-SCa enhanced the expression of anti-inflammatory genes and reduced intestinal pro-inflammation linked to immunological function (Figure 2). The enhancement of villi growth and tight junction can be explained for the incretion of laying hen performance. The results correlate with Chen et al. (2022), that study on intestinal morphology and barrier function in broiler chickens. Intestinal performance, feed nutrient absorption function, and productivity in laying hens are enhanced by villus growth and barrier function (Nii, 2022; Lu et al., 2023). The diet enriched with BSFLO-SCa has a significant amount of lauric acid, as shown in Table 3. Birds given feed containing lauric acid showed enhanced productivity, immunological response, and digestive system function, such as villus growth and barrier function (Wu et al., 2021).

### Jejunal Histomorphology

The structural design of the small intestine is crucial for the efficient digestion and absorption of nutrients. Any changes in the intestinal morphology of the villi can directly affect the body's ability to absorb nutrients effectively (Cui et al., 2019). Villus height, crypt depth, and the villus-to-crypt ratio are used for assessing the mucosal morphology of the small intestine, as well as determining the absorptive capacity of the small intestine (Greig and Cowles, 2017). In the current study, dietary supplementation of 1% BSFLO-SCa increased the intestinal villus height and villus width, while crypt depth increased with 2% BSFLO-SCa. In line with current finding, previous studies reported that the supplementation of BSFLO increased the villus height, but the same studies showed no effect on villus width, and crypt depth (Kim et al., 2021; Chen et al., 2022). Contrast, Schiavone et al. (2018) and Spranghers et al. (2018) reported that supplementation of BSFLO did not affect intestinal histomorphology. The reason for this was unclear, the improvement of intestinal histomorphology in the current study be associated with lauric acid content of BSFLO-SCa. Medium chain fatty acids, such as lauric (C12:0) and myristic (C14:0) stimulate the renewal of intestinal epithelial cells and in turn lead to increased villi length. MCFA are absorbed directly by the intestinal epithelial cells and utilized directly by the enterocytes for energy production and thereby help to support the villi growth hence improved the integrity of the intestinal tissue (Zeitz et al., 2015). Moreover, lauric acid has antibacterial characteristics against Gram-positive bacteria by causing destabilization in bacterial cell walls and cytoplasmic membranes upon integration, resulting in improved the intestinal membrane instability (Zheng et al., 2023). The inclusion of 1% BSFLO-SCa in diet of this study can be assumed as the optimum concentration for increasing lauric acid, which enhances the villus growth. Further doubling of the inclusion will no longer increase the villus growth.

### Tight Junction Gene Expression

Polyunsaturated fatty acids (**PUFA**), mainly linoleic acid, affect the activity of transcription factors that control the expression of tight junction genes such as CLDN-1 and OCLN, as the result contribute the integrity and permeability of epithelial barrier (Xu et al., 2021). The PUFA content of diet supplemented by BSFLO-SCa was dominated by linoleic acid (C18:2), which can be associated with the enhancement of tight junction gene expression, considerably increased the expression of CLDN-1, OCLN, and JAM-2 genes. Previous research by Xu et al. (2021) found that supplementing pigeon squabs with 1% linoleic acid led to increased expression of the CLDN-3 and OCLN genes. In broiler chickens, administration of orally ingested carvacrol essential oil that contains high linoleic acid, resulted in increased expression of the OCLN, CLDN-1, and CLDN-5 genes (Gilani et al., 2018). In the present study, the upregulation of tight junction gene expression may be associated with increased in linoleic acid resulting from BSFLO-SCa supplementation in diet (Table 2).

Linoleic acid acts as a precursor for synthesizing PUFAs, such as n-6 PUFAs, an essentials component of the membrane phospholipids. This membrane is important for facilitating the movement of these substances through the cell membrane so that the process of absorbing nutrients takes place efficiently, and it also plays a role in protecting the body from invasion by pathogens such as bacteria and viruses (Ballweg and Ernst, 2017). The inclusion of linoleic acid in phospholipids can alter the fatty acid composition of the membrane and affect its biophysical properties, leading to modifications in membrane fluidity. Additionally, linoleic acid can influence the function of membrane proteins, including CLDN and OCLN by suppressing transcription factors such as AP-1, NF-kappaB, and SP-1, which have been implicated in regulating. These proteins play a crucial part in fortifying tight junctions responsible for maintaining the integrity and barrier function of the epithelial cell membrane. Alterations in membrane composition and fluidity resulting from linoleic acid may have an impact on the activity of CLDN and OCLN, thus potentially impacting cellular processes that are regulated by tight junctions (Maulucci et al., 2016).

Linoleic acid can activate signaling pathways that affect gene expression, particularly those involved in inflammatory responses and gene regulation. It has been demonstrated that linoleic acid activates the transcription factor nuclear factor-kappaB (**NF-kappaB**) in endothelial cells, resulting in the expression of proinflammatory molecules like vascular adhesion molecule-1 (**VCAM-1**) and plasminogen activator inhibitor-1 (**PAI-1**). Furthermore, the introduction of linoleic acid into endothelial cells can lead to an elevation in oxidative stress, activate NF-kappaB, and have an impact on the levels of interleukin-8 (**IL-8**) and intercellular adhesion molecule-1 (**ICAM-1**). On the other hand, in macrophages, linoleic acid can modulate lipopolysaccharide (**LPS**)-induced inflammatory responses by inhibiting NF-kappaB activation and reducing the expression of inducible nitric oxide synthase (**iNOS**) and cyclooxygenase 2 (**COX2**) with prevents phosphorylation of the inhibitor protein, IkappaB, thus blocking the translocation of NF-kappaB into the nucleus so inhibit inflammatory gene expression or directly interfere with the DNA binding activity of NF-kappaB and suppressing its transctiptional activity (Dichtl et al., 2002; Cheng et al., 2004; Suh et al., 2008). The available evidence indicates that linoleic acid can inhibit the signaling pathways involved in regulating gene expression and the inflammatory response, such as NF-kappaB, as the result decrease the pro-inflammatory gene expression such as IL-18.

Furthermore, the BSFLO-SCa contains MCFAs, specifically lauric acid, that possess anti-inflammatory properties capable of reducing intestinal inflammation, thus enhancing the integrity of intestinal tight junctions. The study demonstrated a reduction in TNF-α, TLR-4, IL-18, and IL-6 gene expressions upon inclusion of BSFLO-SCa in the feed (Figure 2). When inflammatory cytokines like TNF-α is reduced, the activity of the basolateral Na+-K+-2Cl- cotransporter (**NKCC1**) also decreases, impacting paracellular ion transportation and epithelial barrier function. This results in the downregulation of tight junction protein expression and decreased paracellular ion flow (Koumangoye et al., 2022).

### Inflammation Gene Expression

Fatty acid supplementation can modulate the immune system in chickens through both cellular and humoral immune responses, influencing proliferation, maturation, function, and cytokine production of lymphocytes, heterophils, and splenocytes (Alagawany et al., 2019). Based on the results of the current study, the lauric acid content of the 2% BSFLO-SCa diet had the highest value compared to other treatments (Table 3). Lauric acid has been found to play a role in the immune system of chickens by influencing their growth, immune functions, and gut health. Studies have shown that lauric acid supplementation can promote broiler growth and immune functions by regulating lipid metabolism and gut microbiota (Wu et al., 2021). Additionally, a combination of lauric acid monoglyceride and cinnamaldehyde has been found to improve production, intestinal health, and antibacterial properties of broiler chickens (Zheng et al., 2023). Lauric acid, a major component of black soldier fly (**BSF**) oil, has been found to have immunomodulatory potential and can influence the immune system in various ways (Borrelli et al., 2021; Koutsos et al., 2022). Lauric acid has been shown to suppress TLR4-mediated proinflammatory cytokines in macrophages and modulate TLR2-mediated proinflammatory cytokines expression and secretion in macrophages (Richter et al., 2023). In particular, BSFLO contains 40 to 50% lauric acid, a saturated medium-chain fatty acid (**MCFA**) that has been shown to have anti-inflammatory properties (Richter et al., 2023). Therefore, the dietary treatment reduced the expression of pro-inflammatory genes. As a result, anti-inflammatory gene expression was increased.

Medium chain fatty acids have unique abilities to modulate immunity. It has been noticed that MCFA-enriched diets, as opposed to those supplemented with long chain fatty acids (Papada et al., 2014), lard (Geng et al., 2016) or omega-6 enriched formulations (Bertevello et al., 2012), decrease inflammation in animal models of dextran sulfate sodium (**DSS**)-induced colitis. *In vivo* evidence of the inhibitory crosstalk between C12:0 and TLR/NF-κB signaling was found in rats with liver inflammation generated by LPS (Khan et al., 2021) and mice with ear edema induced by P. acnes (Huang et al., 2014). Furthermore, it was shown that MCFAs increased the expression of secretory immunoglobulin A (**IgA**) in the rat gut in response to LPS (Kono et al., 2004). Secreted IgA has been shown to have a therapeutic impact on animals with colitis models and is essential for the body's defense against infections (Okai et al., 2016). Black soldier fly larvae oil has monounsaturated palmitoleic (C16:1) and oleic (C18:1) acids in addition to C12:0. According to earlier research, these fatty acids improved the variety of the gut microbiota in both healthy and sick animal models, which lessens the symptoms of Inflammatory Bowel Disease (**IBD**) (Machate et al., 2020). Furthermore, the inclusion of BSFLO-SCa reduced palmitic acid (C16:0) content in diet, with the supplementation of 2% BSFLO-SCa resulting in the lowest levels (Table 3). Korbecki and Bajdak-Rusinek (2019) reported that palmitic acid has been extensively studied or its role in inducing inflammation. Palmitic acid has been shown to strengthen TLR-4 induce signaling with 2 mechanisms. Lu et al. (2017) explained that palmitic acid can directly bind to activate TLR-4 by matching the structure of LPS, allowing it to bind and activate TLR-4 similarly to LPS. Additionally, palmitic acid also activates TLR-4 indirectly by inducing cellular stress, such as endoplasmic reticulum stress and mitochondrial dysfunction, leading to the production of reactive oxygen species (**ROS**) and other inflammatory mediators, as the results trigger activation of the TLR-4 signalling pathway and contributing to inflammation. The decreased inflammation in this study can be explained by the reduction of palmitic acid with the inclusion of BSLF-SCa.

## CONCLUSIONS

In conclusion, dietary supplementation of 2% BSFLO-SCa in laying hens improved production performance (HDA, EM, EW, FCR), intestinal histomorphology (VH, VW, and CD) and integrity by upregulating tight junction-related protein gene expression (CLDN-1, OCLN, and JAM-2). In addition, BSFLO-SCa improved intestinal health through modulation of jejunal immune responses and inflammatory cytokine gene expressions.
